# The contribution of the anaesthetist to risk‐adjusted mortality after cardiac surgery†

**DOI:** 10.1111/anae.13291

**Published:** 2015-10-28

**Authors:** O. Papachristofi, L. D. Sharples, J. H. Mackay, S. A. M. Nashef, S. N. Fletcher, A. A. Klein, the Association of Cardiothoracic Anaesthetists (ACTA), G Lau, D Woodward, J Hillier, M Ware, S Agarwal, M Bill, R Gill, D Duthie, H Skinner

**Affiliations:** ^1^ MRC Biostatistics UnitInstitute of Public HealthCambridgeUK; ^2^ Clinical Trials Research UnitUniversity of LeedsLeedsUK; ^3^ Department of Anaesthesia and Intensive CarePapworth HospitalCambridgeUK; ^4^ Department of Cardiothoracic SurgeryPapworth HospitalCambridgeUK; ^5^ Departments of Anaesthesia and Critical CareSt Georges University HospitalLondonUK

## Abstract

It is widely accepted that the performance of the operating surgeon affects outcomes, and this has led to the publication of surgical results in the public domain. However, the effect of other members of the multidisciplinary team is unknown. We studied the effect of the anaesthetist on mortality after cardiac surgery by analysing data collected prospectively over ten years of consecutive cardiac surgical cases from ten UK centres. Casemix‐adjusted outcomes were analysed in models that included random‐effects for centre, surgeon and anaesthetist. All cardiac surgical operations for which the EuroSCORE model is appropriate were included, and the primary outcome was in‐hospital death up to three months postoperatively. A total of 110 769 cardiac surgical procedures conducted between April 2002 and March 2012 were studied, which included 127 consultant surgeons and 190 consultant anaesthetists. The overwhelming factor associated with outcome was patient risk, accounting for 95.75% of the variation for in‐hospital mortality. The impact of the surgeon was moderate (intra‐class correlation coefficient 4.00% for mortality), and the impact of the anaesthetist was negligible (0.25%). There was no significant effect of anaesthetist volume above ten cases per year. We conclude that mortality after cardiac surgery is primarily determined by the patient, with small but significant differences between surgeons. Anaesthetists did not appear to affect mortality. These findings do not support public disclosure of cardiac anaesthetists' results, but substantially validate current UK cardiac anaesthetic training and practice. Further research is required to establish the potential effects of very low anaesthetic caseloads and the effect of cardiac anaesthetists on patient morbidity.

## Introduction

It is accepted that the operating surgeon may affect risk‐adjusted mortality following cardiac surgery, and this has led to the publication of surgeon‐specific mortality rates in the UK and elsewhere (see http://www.scts.org/patients/hospitals/) 1, 2. The fact that cardiac surgery is undertaken by teams has inevitably led to the suggestion that other team members – notably the anaesthetist – should be subject to similar scrutiny, and that anaesthetist‐specific, risk‐adjusted outcomes should be similarly available 3, 4, 5, 6.

Objective evaluation of the contribution of individual anaesthetists to postoperative outcome is difficult. A link between the individual anaesthetist and outcomes (myocardial ischaemia and infarction) was suggested 30 years ago in a landmark study by Slogoff and Keats 7. Merry et al. demonstrated a potential link between patient outcome and individual anaesthetists 8, but the topic received scant attention over the next two decades. Two recent attempts to assess the impact of the anaesthetist on cardiac surgical outcomes have produced conflicting results. A single‐centre UK study of 18 662 patients found that the individual anaesthetist had a minimal impact on risk‐adjusted mortality 9. In contrast, a North American retrospective observational study of 7920 patients, based on prospectively collected data from the New York State Cardiac Surgery Reporting System, found evidence of substantial variability in death or major complications between anaesthetists 10. A possible explanation for the apparent transatlantic differences in the impact of the anaesthetist is the difference in anaesthetic practice. In the UK study centre, anaesthetists' workload was entirely cardiothoracic, largely protocol‐driven and cardiac caseload was high. In contrast, in the North American study, many anaesthetists had mixed practices, lower annual cardiac caseloads and greater variation in protocols.

In surgery, mortality may be inversely related to caseload volume 11, 12. The analogous impact of anaesthetic caseload volume is unexplored. Cardiothoracic anaesthesia and intensive care has developed into a sub‐speciality in its own right, and this has led to a debate as to whether anaesthetists should also undertake a minimum annual caseload.

We were motivated by the hypotheses that there may be variation in cardiac surgical outcomes between anaesthetists as there is between surgeons, and that caseload volume may be associated with patient outcomes. Hence, the aim of our study was to assess the anaesthetists' impact on the variation in outcomes after cardiac surgery and to establish whether caseload volume may affect patient outcome.

## Methods

**Figure 1:**
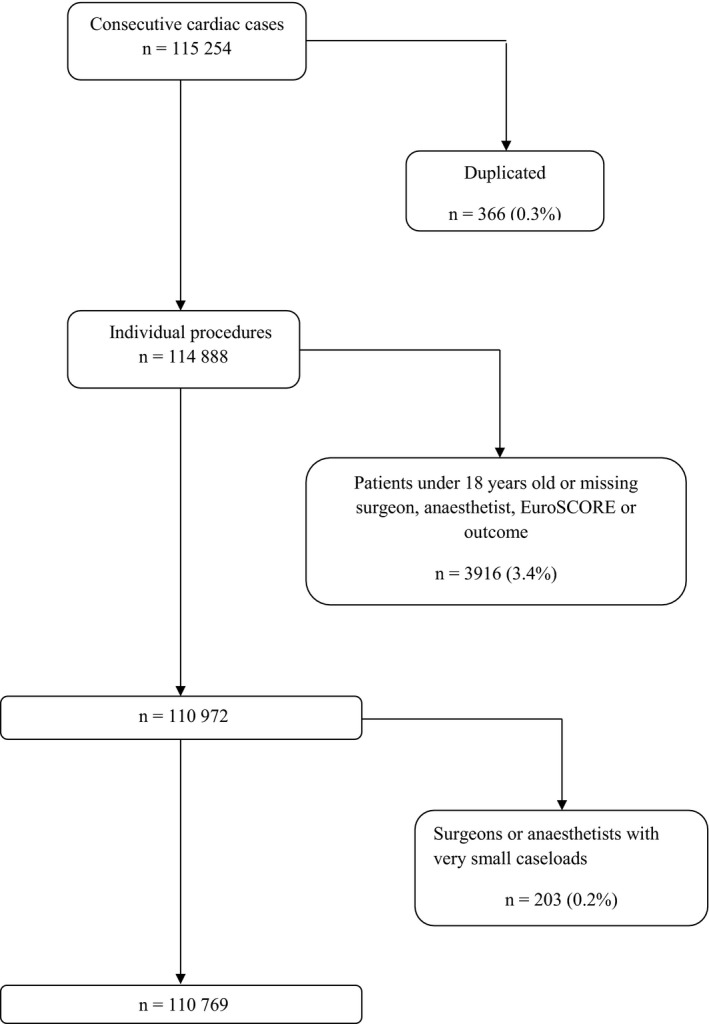
Flow diagram of participants.

All 36 UK specialist cardiac surgical centres were invited to take part in the study; a time frame of one month was given to respond and to secure relevant permissions. Of the 36 centres, ten volunteered for participation (Bristol University Hospital, Cardiff University Hospital, City Hospital Nottingham, Glenfield Hospital Leicester, Leeds General Infirmary, Liverpool Heart and Chest Hospital, Northern General Hospital Sheffield, Papworth Hospital Cambridge, Royal Victoria Hospital Belfast, and Southampton University Hospital), and obtained the relevant local permissions for data collection within the set time frame. The requirement for formal ethical approval was waived according to the National Research Ethics Service of the NHS Health Research Authority. All centres collected data prospectively as part of NHS requirements and provided these data to the Society of Cardiothoracic Surgeons and National Institute for Cardiovascular Outcomes Research; these datasets were then provided to the Association of Cardiothoracic Anaesthetists (ACTA) in 2014. Data from consecutive major cardiac operations were prospectively collected for the period April 2002 through March 2012 (Fig. 1), with the exception of centre no. 4 (April 2002 through March 2013), and centre no. 8 (April 2004 through August 2013). Cardiac transplants, pulmonary endarterectomy procedures, very high‐risk cases that required operation by two or more consultant surgeons, and other procedures for which the logistic EuroSCORE 13 was not suitable, were not studied. Patients under 18 years of age were also not studied.

The primary outcome measure was in‐hospital death up to three months postoperatively; patients who were transferred out of the hospital in which they had their surgery to another hospital were considered to have survived. The logistic EuroSCORE was used to adjust for different patient casemix; this is a very well‐established risk score, given as a percentage, specifically constructed to be used as a risk predictor for in‐hospital death after cardiac operations. It includes 17 cardiac, operation‐ and patient‐related factors and is used for risk assessment in many countries. This is the principal patient covariate we considered and it should be sufficient since all important patient‐related factors for in‐hospital mortality were included in its construction, with appropriate weighting 13, 14. Although the original logistic EuroSCORE 13 has been recalibrated (EuroSCORE‐2 14), the original version was in use during this study and was the version supplied by participating centres. The primary covariate of interest was the caseload volume of anaesthetists and surgeons.

We used logistic random‐effects regression analysis 15, 16, 17 to analyse the relationship between in‐hospital death and potential covariates. The response was death within three months of the procedure. Our analysis reflected the hierarchical nature of the data (patients grouped within surgeons/anaesthetists who are grouped within centres) using ‘random‐effects' for centres, surgeons, and anaesthetists. The logistic EuroSCORE was included as a fixed effect in all models to standardise for different patient risk‐profiles; this was achieved by dividing the scores by 100 to transform them to probabilities and taking their logit transform.

We first fitted two three‐level, random intercept models to establish the effects of individual surgeon and anaesthetist on the patient outcome, controlling for centre effects and casemix risk. To investigate the combined effects of surgeon and anaesthetist, we fitted a three‐level, cross‐classified model assuming an additive, individual contribution from each provider (anaesthetist and surgeon), nested with centres. To investigate the effect of volume on outcome, we refitted the three‐level, cross‐classified model including the monthly average volume of cases per surgeon and anaesthetist, defined as the total number of operations performed divided by the number of months in active practice. For each model, the intra‐class correlation coefficients (ICC) 17 were estimated, interpreted as the proportion of the total variation that can be attributed to each of the anaesthetist, surgeon and centre. The p values determining the significance of the fixed‐effect terms were calculated using the likelihood ratio test. Analyses were implemented using R (version 3.0.1, see http://www.r-project.org) 18, 19.

When reporting the results, we have not provided 95% CI. Confidence intervals for the proportion of the variation explained by different components in a hierarchical dataset are extraordinarily difficult to estimate. The technical statistical derivation has not been published (to our knowledge). We can never show that a variance component is zero, or even that a CI includes zero. Software does not normally provide standard errors for the random‐effects variance either, and only a likelihood ratio test is recommended to judge the significance of the random‐effects terms.

## Results

There were missing outcomes of interest, for which records could not be retrieved, in three centres. Since the proportions of missing data from these three centres were very small (0.01%, 0.01% and 1.5% of n = 9900, 18 515 and 7793, respectively), we removed cases with missing outcome from the dataset. In four centres, a small number of missing surgeon entries were found (0.01%, 0.04%, 0.02% and 0.2% of n = 15 461, 7793, 9900 and 6903, respectively), and these were excluded from the analysis. Eight of the ten centres had missing anaesthetist entries, with the largest proportion reaching 3% in centre no. 6 (n = 9900); the percentages in other centres varied from 0.1% to 1.5%. Since the anaesthetist could not be informatively imputed and these percentages were small, these cases were excluded. Finally, missing EuroSCORE entries from five centres (0.03%, 0.7%, 1.2%, 1.9% and 5.0% of n = 6625, 9900, 9633, 7501 and 7793, respectively) were removed from the data.

In all centres, surgeons and anaesthetists who each performed < 0.1% of the cases in their centre were excluded; this was fewer than 10 operations per professional except for one surgeon. These professionals had either retired just after the start of the study period, were appointed just before the end of the study period, or held short‐term contracts at their centre.

Final analysis was performed on 110 769 cases after exclusions, 96% of the original case series of 115 254 patients, treated by 127 surgeons and 190 anaesthetists in ten centres. The analysis was done using 91% (127/140) and 76% (190/250) of the original surgeon and anaesthetist samples, respectively, mostly due to the low‐volume exclusions. Baseline characteristics for the study cohort are summarised in Table 1. Overall, 3413 of 110 769 patients (3.1%) died in‐hospital. In‐hospital mortality for the subset of professionals with very small caseloads was comparable with mortality in individual centres (3.45% of n = 203, see Table 2) as well as overall mortality for this dataset. The cases performed in each centre are summarised in Table 2, together with death rates, EuroSCORE and number of surgeons and anaesthetists per centre. For one centre, the additive EuroSCORE was provided, which leads to under‐prediction in high‐risk patients. Sensitivity analysis excluding this centre did not differ from the analysis including it. The proportion of patients lying above the risk level where the additive EuroSCORE starts to underperform (EuroSCORE ≥ 10%) was very small (0.62%, n = 689 of 110 769) 14, 20. All centres were high‐volume, with only two having fewer than 800 cases per year, the largest high‐volume threshold encountered in the literature 21. All centres exceeded the 400 cases threshold recommended for cardiac operations by the American Heart Association (AHA).

**Table 1:** Characteristics of cardiac surgical patients and procedure performed (n = 110 769). Values are mean (SD) or number (proportion)

Age at admission; years	66.4 (11.3)
Logistic EuroSCORE; %	7.36 (9.88)
Male	80 603 (72.8%)
Priority	
Elective	76 540 (69.1%)
Urgent	29 646 (26.8%)
Emergency	4123 (3.7%)
Salvage	419 (0.4%)
Unknown	41 (0.04%)
Operation type	
Isolated CABG	57 644 (52.0%)
Isolated AVR	9956 (9.0%)
MVR	6475 (5.8%)
CABG + AVR	9050 (8.2%)
CABG + other	5466 (5.0%)
Other procedure	16 000 (14.4%)
Unknown	6178 (5.6%)

CABG, coronary artery bypass grafting; AVR, aortic valve replacement or repair; MVR, mitral valve replacement or repair.

**Table 2:** Numbers of patients operated on and surgeons and anaesthetists in each centre, between April 2002 and March 2012. Surgeons and anaesthetists who looked after < 10 patients per year were excluded. Values are number or mean (SD)

Centre no.	Patients	Surgeons	Anaesthetists	Deaths	Mortality	Logistic EuroSCORE
1	18 515	21	24	575	3.11%	8.07 (10.77)%
2	9633	13	16	273	2.83%	9.48 (12.26)%
3	6625	6	8	247	3.73%	8.23 (10.18)%
4	15 461	16	24	449	2.90%	6.16 (8.15)%
5	6907	10	15	220	3.19%	6.61 (9.00)%
6^a^	9900	10	17	243	2.45%	4.42 (3.35)%
7	7793	13	17	219	2.81%	7.99 (11.47)%
8	7501	11	13	215	2.87%	7.21 (10.91)%
9	17 112	17	22	577	3.37%	7.98 (10.54)%
10	11 322	10	34	395	3.49%	7.28 (8.58)%

a Additive EuroSCORE was provided by this centre (see text).

The yearly caseload varied considerably among surgeons and anaesthetists, both between and within centres. Nevertheless, most surgeons (104/127, 81.9%) can be considered high‐volume as they performed more than the 75 operations per year recommended by the AHA. Likewise, most anaesthetists (150/190, 79%) anaesthetised for more than 50 operations per year.

The logistic EuroSCORE was a significant covariate in both the three‐level surgeon and anaesthetist models for the in‐hospital mortality outcome, adjusted for the centre (OR 0.903 (95% CI 0.875–0.931) and 0.896 (95% CI 0.869–0.924), respectively; p value < 0.0001 for both). The logistic EuroSCORE remained significant in the three‐level, cross‐classified model adjusting for the surgeon and anaesthetist simultaneously (OR 0.903 (95% CI 0.876–0.930; p value < 0.0001). The proportion of the variation in in‐hospital death attributed to EuroSCORE (and other unexplained variables) from the three‐level, cross‐classified model was 95.75% (Table 3).

**Table 3:** Variation in in‐hospital death attributed to each group. Values are proportion

Centre	Surgeon	Anaesthetist	Patient and other covariates
0%	4.00%	0.25%	95.75%

**Figure 2:**
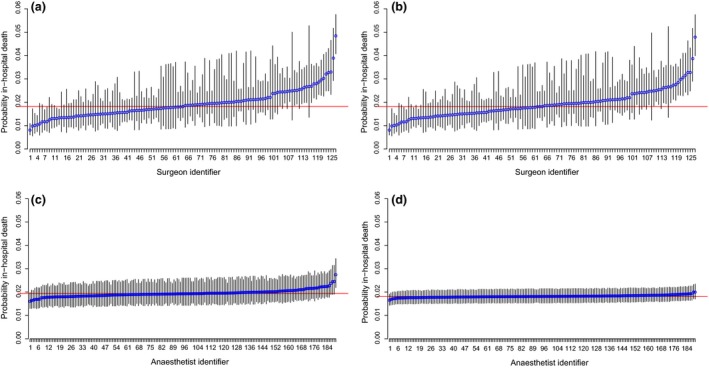
Estimated probability of in‐hospital death within three months of surgery for a patient with average EuroSCORE risk: (a) surgeons adjusted for centre only; (b) surgeons adjusted for centre and anaesthetist; (c) anaesthetists adjusted for centre only; (d) anaesthetists adjusted for centre and surgeon. The horizontal line is average probability (1.8%) for the study cohort. Error bars = 95% CI.

Figure 2 shows the estimated probability of in‐hospital death for each surgeon if they operated on a patient with the mean EuroSCORE (estimated at 7.4%), controlling for the centre effect only, and controlling for both the centre and anaesthetist effects simultaneously. Estimated probabilities of death for eight out of 127 surgeons, from four different centres, have their 95% CI lying wholly below the average probability of death, indicating low mortality. There were 19 surgeons from nine centres whose estimated probability of death was higher than average. The surgeon random‐effects variance was moderate but significant with ICC_surgeon_ = 0.0406, suggesting that 4.06% of the variation in outcome was attributable to the operating surgeon (Table 3). Adjusting for anaesthetist did not have an effect on the surgeon plots and reduced ICC_surgeon_ slightly from 0.0406 to 0.0400, indicating that the operating anaesthetist's impact on the outcome is minimal compared with that of the surgeon. After adjusting for surgeon effects, there were no remaining centre effects.

From the centre‐anaesthetist model that adjusted for patient risk, the anaesthetist random‐effects variance was very small (ICC_anaesthetist_ = 0.0071). Figure 2c demonstrates that there is almost no between‐anaesthetist variability in the outcome, with only one anaesthetist performing significantly differently from the average. In the cross‐classification model adjusting also for surgeon effects, anaesthetist variation reduced to ICC_anaesthetist_ = 0.0025 which is negligible (Fig. 2d), with no anaesthetist significantly different from the average. The ‘outlying' anaesthetist in Fig. 2c performed 73% of his cases with the ‘worst' performing surgeon in his centre; it is thus possible that his results were driven by the surgeon with whom he/she principally worked, thus falsely appearing suboptimal compared with the other anaesthetists. Once we adjusted for the surgeon as well as the anaesthetist in the three‐level, cross‐classified model, the impact of the surgeon on the anaesthetist's performance was accounted for and the specific anaesthetist was no longer significantly different from average (Fig. 2d). The difference in the probability of in‐hospital death between the two anaesthetists at the extremes reduced from about 1.5% to 0.5%.

With respect to both surgeons and anaesthetists, there was a weak association between increased volume of cases performed and reduction in mortality, OR 0.99 (95% CI 0.96–1.01; p = 0.277) and 0.99 (95% CI 0.98–1.01; p = 0.217), respectively (see Supporting Information, Appendix S1).

## Discussion

Our study cohort of 110 769 patients is the largest study to date of the impact of individual anaesthetists on patient outcome. This study includes data from ten of the 36 UK cardiothoracic surgical centres and incorporates almost a third of all UK cardiac operations undertaken during the one‐decade study period. Patient risk accounted for 95.75% of the variation in in‐hospital mortality. The second largest effect can be ascribed to the surgeon (Table 3 based on the risk‐adjusted model adjusting for centre, surgeon and anaesthetist). Adjusting for the anaesthetist and centre components, the surgeon accounted for 4.00% of the observed variation in‐hospital mortality. In comparison, the variation in mortality explained by the individual anaesthetist was minimal (0.25%). There was no remaining variation attributable to the centre.

Our key findings from a heterogeneous group of ten UK centres were very similar to our previously reported findings from a single, large, specialist cardiothoracic hospital 9. Surgeons had a small but measurable effect on outcome, whereas no effect was found for the anaesthetist. A literature review identified only one other recent publication assessing the effect of the anaesthetist on cardiac surgical outcomes. In contrast to our findings, Glance et al. reported significant variation in performance between anaesthetists 10. Different statistical methodology, study design and surgical practices could account for these conflicting findings. Glance et al. used a fixed‐effects model, which may not have accounted for the simultaneous effects of the surgeon and the centre. In UK clinical practice, it is usually found that pairings between surgeons and anaesthetists are not random, as they most often are in the USA as reported by Glance et al. As shown by our study, it is possible that part of the variation attributed to the anaesthetist could be explained by the operating surgeon, accounted for in our methodology by using random‐effects modelling accounting for all centre, surgeon and anaesthetist groupings simultaneously. The principal advantage of our methodology is that it allows the anaesthetist, surgeon or centre to be treated as a random sample from the whole population; that is, if we had chosen 190 other anaesthetists, the distribution of their results would have been similar, yielding generalisable estimates 22. In contrast, fixed‐effects models restrict results only to the sample of anaesthetists (surgeons or centres) available. Failure to take the dependency between each group's patients into account during analysis may result in bias in the estimated group and covariate effects, and inaccuracy in their respective standard errors and p values 17. Our approach also allows us to delineate operator average effects from the effect of their caseload volume. Differences in outcome measurements, anaesthetic practice, training and size of surgical centre are additional factors that could explain the differences in findings. Glance et al. used a composite outcome of in‐hospital mortality (no measurement period specified) and other major complications on which the anaesthetist may be more influential. No known risk score for this outcome is available, although Glance et al. included several risk factors in their analysis in order to adjust for differences in casemix. Anaesthetic care may be more standardised in UK centres than in the USA, thus allowing less scope for variation in practice to be observed. In the UK, consultants undertaking cardiothoracic anaesthesia have almost invariably undertaken additional sub‐speciality training. Although this is also the case for US anaesthetists working in large surgical centres, this may not be the case in many of the smaller US surgical centres.

This study suggests that the standard of cardiac anaesthetic care in the ten UK centres studied is consistently high, but we acknowledge that these findings may not apply elsewhere in the UK or worldwide. Our study has demonstrated a robust mechanism for detecting underperformance, and we recommend that it should be applied to all UK centres with an interest in the monitoring of anaesthetic performance 23.

Perhaps surprisingly, we found no evidence of variation due to the centre. One potential limitation is the possibility that centres that volunteered to participate were different, in terms of patient risk treated or between‐provider variability, from those opting not to participate. It is possible that the small number of participating centres and the potential bias due to their self‐selection may have resulted in underestimation of the centre variation in our study. Furthermore, any variation in centre performance might be accounted for solely by variation in surgeon performance. Moreover, there is increasing evidence that anaesthetic care may affect patient outcomes such as major postoperative complications (e.g. stroke and myocardial infarction) 24. A further limitation of our study is that we did not consider such composite outcomes and we underline the need for large studies on these to obtain robust evidence of the relative impact of the anaesthetist. The study was conducted in UK specialist cardiothoracic centres, where anaesthetic practices are often protocol‐driven; this limits the potential for variation in the standard of care. Therefore, the findings may not apply outside of the UK where practice may differ. There was a small percentage (< 3.4%) of missing data in our dataset, which occurred mostly at the start and end of the recorded series. Blocks of missing data at the end of series are likely to have been due to delays between completed hospital episodes and data entry on to hospital electronic data systems. Moreover, in some centres, the consistent recording of the logistic EuroSCORE was not in place from the start of the series (in 2002), resulting to some missing data. In both these cases, missing data can be described as due to administrative reasons and assumed to be missing completely at random. Finally, in centre 6, one of the participating surgeons omitted to record the specific anaesthetist with whom he was principally working, resulting in missing anaesthetist data; hence, we excluded these records from further analysis. A sensitivity analysis including this surgeon and imputing his missing anaesthetist entries did not alter the results. Professionals with very small caseloads were excluded from analysis to avoid problems with model fit due zero events. However, as the exclusion of low‐volume professionals resulted in few exclusions (0.2%) and, since mortality in this subset was comparable with that of the full dataset (3.45% and 3.1%), it is unlikely that this induced bias in the results.

This study was embarked upon by ACTA primarily to answer two questions: (i) should individual anaesthetists' outcomes be published on the Internet? and (ii) what is the safe minimum annual caseload? Based on our findings, the answer to the first question is a resounding ‘no' in the UK. Publication of these results appears unnecessary and may have unintended consequences, such as avoidance of high‐risk cases, already observed in cardiac surgical practice 25.

The second question is currently more difficult to answer. Our study suggests that performance is consistent in anaesthetists who complete at least ten cases per year and the second question is partially unresolved. Separate subgroup analysis of the combined outcomes of our very low‐volume UK colleagues is probably required to answer this question. Although there was a weak association between higher monthly case volume and survival, our results suggest that caseload may be less important than previously thought. Increased morbidity (rather than death) associated with low annual case volumes may be an additional reason for Glance's et al.'s apparent conflicting findings.

In conclusion, in the ten UK specialist centres studied, the overwhelming factor associated with in‐hospital mortality was the patients' risk profile, with the individual surgeon having a small but statistically significant contribution to variation in mortality. The impact of the individual anaesthetist was minimal. The operating centre did not have an effect on the outcome. We propose that this study substantially validates current UK specialist training and practice in cardiothoracic anaesthesia as fit for purpose, at least as far as it affects patient mortality. We recommend that further study to examine the effect of cardiac anaesthetists on patient morbidity be carried out.

## Competing interests

No other competing interests declared.

## Supporting information


**Appendix S1.** Model outputs and construction of Table 3.

## Appendix 1

### Collaborators

G Lau, Glenfield Hospital, Leicester, UK

D Woodward, Northern General Hospital, Sheffield, UK

J Hillier, University Hospitals, Bristol, UK

M Ware, University Hospital of Wales, Cardiff, UK

S Agarwal, Liverpool Heart and Chest Hospital, Liverpool, UK

M Bill, Royal Victoria Hospital, Belfast, UK

R Gill, University Hospital, Southampton, UK

D Duthie, Leeds General Infirmary, Leeds, UK

H Skinner, City Hospital, Nottingham, UK
