# Supplementary material for: The contribution of the anaesthetist to risk‐adjusted mortality after cardiac surgery
Source: Anaesthesia. 2015 Oct 28;71(2):138–46. doi: 10.1111/anae.13291 (PMC4949638; doi:10.1111/anae.13291)

**Supporting Information (online only)**

**Appendix 2**

**Table A1** Model output for the random intercept three-level surgeon model. Values are number (95% CI) or number.

|  | **Estimate** | **p value** |
| --- | --- | --- |
| *Fixed effects* |  |  |
| Model Intercept | –4.01 (–4.11 to –3.91) | *<* 0*.*0001 |
| Logistic EuroSCORE | 0.903 (0.875–0.931) | *<* 0*.*0001 |
|  |  |  |
| *Random effects* | **Centre variability** | **Surgeon variability** |
|  | 8.15x10^-13^ | 0.139 |

**Table A2** Model output for the random intercept three-level anaesthetist model. Values are number (95% CI) or number.

|  | **Estimate** | **p value** |
| --- | --- | --- |
| *Fixed effects* |  |  |
| Model Intercept | –4.01 (–4.11 to –3.91) | *<* 0*.*0001 |
| Logistic EuroSCORE | 0.896 (0.869 – 0.924) | *<* 0*.*0001 |
|  |  |  |
| *Random effects* | **Centre variability** | **Anaesthetist variability** |
|  | 0.0250 | 0.0243 |

**Table A3** Model output for the random intercept three-level cross-classified model. Values are number (95% CI) or number.

|  | **Estimate** |  | **p value** |
| --- | --- | --- | --- |
| *Fixed effects* |  |  |  |
| Model Intercept | –4.01 (–4.11 to –3.91) |  | *<* 0*.*0001 |
| Logistic EuroSCORE | 0.903 (0.876–0.930) |  | *<* 0*.*0001 |
|  |  |  |  |
| *Random effects* | **Centre variability** | **Surgeon variability** | **Anaesthetist variability** |
|  | 8.27x10^-9^ | 0.138 | 0.00854 |

*Construction of Table 3*

We used the random effects variance estimates from Table 3 in order to compute the variation in in-hospital death attributed to each group. The formula for a three-level cross-classified model is given by [18]:

$${ICC}_{A}=\frac{{variance}_{A}}{{variance}_{A}+{variance}_{B}+{variance}_{C}+\pi^{2}/3}$$

For the three-level random intercept models, the respective formula is [18]:

$${ICC}_{A}=\frac{{variance}_{A}}{{variance}_{A}+{variance}_{B}+\pi^{2}/3}$$

Hence, variation due to the surgeon is calculated, using Table A1, as:

$$\frac{0.139}{0.139+8.15\times{10}^{-13}+\pi^{2}/3}=0.0406$$

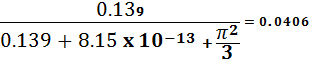

Supplement: Supplementary file 1 — Appendix S1. Model outputs and construction of Table 3. [file ANAE-71-138-s001.docx]
